# Temporal Changes in the Surface Chemistry and Topography of Reactive Ion Plasma-Treated Poly(vinyl alcohol) Alter Endothelialization Potential

**DOI:** 10.1021/acsami.3c16759

**Published:** 2023-12-20

**Authors:** Ryan A. Faase, Novella M. Keeling, Justin S. Plaut, Christian Leycam, Gabriela Acevedo Munares, Monica T. Hinds, Joe E. Baio, Patrick L. Jurney

**Affiliations:** † School of Chemical, Biological, and Environmental Engineering, Oregon State University, 103 Gleeson Hall, Corvallis, Oregon 97331, United States; ‡ Biomedical Engineering Program, University of Colorado Boulder, 1111 Engineering Drive 521 UCB, Boulder, Colorado 80309-0521, United States; § Department of Biomedical Engineering, Oregon Health and Science University, 3303 SW Bond Ave, Portland, Oregon 97239, United States; ∥ Cancer Early Detection Advanced Research Center, Knight Cancer Institute, Oregon Health and Science University, 3303 SW Bond Ave, Portland, Oregon 97239, United States; ⊥ Department of Biomedical Engineering, San José State University, One Washington Square, San Jose, California 95112-3613, United States

**Keywords:** endothelialization, poly(vinyl alcohol), reactive ion plasma, nanotopography, hydrophobic recovery, cardiovascular biomaterials

## Abstract

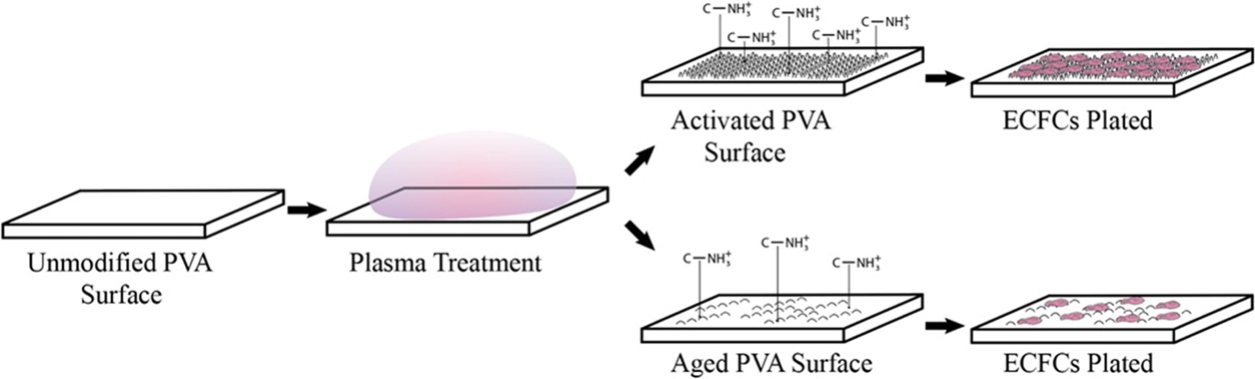

Synthetic small-diameter vascular grafts (<6 mm) are used in the treatment of cardiovascular diseases, including coronary artery disease, but fail much more readily than similar grafts made from autologous vascular tissue. A promising approach to improve the patency rates of synthetic vascular grafts is to promote the adhesion of endothelial cells to the luminal surface of the graft. In this study, we characterized the surface chemical and topographic changes imparted on poly(vinyl alcohol) (PVA), an emerging hydrogel vascular graft material, after exposure to various reactive ion plasma (RIP) surface treatments, how these changes dissipate after storage in a sealed environment at standard temperature and pressure, and the effect of these changes on the adhesion of endothelial colony-forming cells (ECFCs). We showed that RIP treatments including O_2_, N_2_, or Ar at two radiofrequency powers, 50 and 100 W, improved ECFC adhesion compared to untreated PVA and to different degrees for each RIP treatment, but that the topographic and chemical changes responsible for the increased cell affinity dissipate in samples treated and allowed to age for 230 days. We characterized the effect of aging on RIP-treated PVA using an assay to quantify ECFCs on RIP-treated PVA 48 h after seeding, atomic force microscopy to probe surface topography, scanning electron microscopy to visualize surface modifications, and X-ray photoelectron spectroscopy to investigate surface chemistry. Our results show that after treatment at higher RF powers, the surface exhibits increased roughness and greater levels of charged nitrogen species across all precursor gases and that these surface modifications are beneficial for the attachment of ECFCs. This study is important for our understanding of the stability of surface modifications used to promote the adhesion of vascular cells such as ECFCs.

## 1 Introduction

Endothelialization is the process by which endothelial cells (ECs) adhere, migrate, and proliferate to form the endothelial tissue. This process occurs naturally on biological substrates, such as the extracellular matrix (ECM), and can be induced on synthetic materials including polymers and hydrogels. In vivo, endothelial tissue comprises a monolayer of squamous cells lining the internal surfaces of the circulatory and lymphatic systems, firmly anchored to the vessel’s ECM. Clinically, endothelialization is particularly critical for cardiovascular devices like vascular grafts and stents, where it promotes the formation of a compatible endothelial layer on their surfaces.^1^ The presence of endothelial tissue on these devices has been shown to enhance their long-term patency by reducing the risks of thrombosis and restenosis.^2−5^

However, the spontaneous formation of such tissue on synthetic vascular grafts (SVGs), especially those with an inner diameter of less than 6 mm, is an uncommon occurrence, which is a significant factor in their lower patency rates compared to autologous vascular grafts.^6−9^ Despite the clinical preference for autologous grafts in bypass surgeries, they are not without their limitations including donor site morbidity and limited availability. Indeed, an estimated 10–20% of patients requiring a coronary artery bypass graft do not have suitable autologous tissue, often due to factors like varicosities, deep venous thrombosis, prior surgical interventions, or suboptimal vessel quality.^10,11^ For these patients, there is a pressing clinical need for a synthetic biomaterial that can serve as an equivalent or superior alternative to autologous tissue.

A multitude of materials have been explored for their potential to replace autologous vasculature, such as synthetic and natural polymers, decellularized matrices, and self-assembled tissue constructs.^12^ Among these, small-molecule conjugation has emerged as an effective strategy to enhance endothelialization.^13^ Yet, the prevalent molecules designed to attract and support the proliferation of endothelial progenitor cells (EPCs) and ECs face several challenges. They tend to be costly, present difficulties in functionalization onto novel materials, and often lack the necessary structural stability, specificity in capturing target cells, and essential biological functions which together have curtailed their practical applications and clinical viability.^14^

Moreover, while some tissue-engineered vascular grafts (TEVGs) have demonstrated promising results, surpassing autologous grafts or standard SVGs in certain aspects, their high production costs remain a significant barrier to widespread clinical use.^15^ The field stands in need of a surface treatment approach that is not only conducive to endothelialization but also scalable, cost-effective, and nonthrombogenic, to facilitate the clinical adoption of innovative SVG materials.

The inherent surface characteristics of biomaterials, specifically roughness and chemical composition, are paramount in the endothelialization of implanted vascular biomaterials.^16^ Surface roughness, for instance, has been shown to bolster EC adhesion and proliferation, providing essential topographical cues that facilitate cell attachment.^17^ Moreover, the presence of certain functional groups, including carboxylic acids and amino groups, has been recognized for their role in improving cell attachment and proliferation.^18^ Beyond macro-scale textures, nanoscale roughness emerges as a critical feature for modulating EC behavior, further promoting endothelialization across diverse material surfaces.^19^

Research involving polymers like polyethylene reveals that nanoscale surface modifications can substantially improve EC adhesion, spreading, and proliferation.^20^ Advances in biomaterial fabrication techniques, such as electrospinning, nanoimprint lithography, combinatorial drug loading, and enhanced surgical methods, have led to the development of micro- and nanostructured surfaces with precise roughness control. These innovations enable more intricate exploration into the effects of nanoscale features on endothelialization.^21^ The convergence of these findings and technological advancements holds great promise for cardiovascular biomaterial design, suggesting that the integration of nanoscale roughness alongside hydrophilicity and surface charge into biomaterial surfaces could significantly augment endothelialization.

Reactive ion plasma (RIP) treatment is a powerful and versatile technique for creating nanoscale roughness on polymer surfaces. This technique involves the generation of reactive species in a low-pressure plasma environment, which interacts with the polymer surface, altering its chemical composition, topography, and wettability. RIP treatment has been demonstrated to improve the endothelialization of various polymers, including polyurethane (PU), polytetrafluoroethylene (ePTFE), and poly(vinyl alcohol) (PVA).^22−24^ The degree to which the polymeric surface, and therefore its endothelizability, is altered depends on the RIP ion energy, dose, and ion species, which is determined by the precursor gas used to generate plasma.^25^ By modifying surface properties such as roughness and introducing functional groups, RIP treatment can increase the adhesion, migration, and proliferation of ECs on polymer surfaces, thereby promoting endothelialization.

Additionally, RIP treatment provides sterilization benefits, as the reactive species can also eradicate microorganisms, making it a dual-purpose technique.^26^ This feature is particularly advantageous for cardiovascular biomaterials, which necessitate both sterility and enhanced endothelialization potential. However, the efficacy of RIP in promoting endothelialization may be diminished over time. This is due to time-dependent thermodynamically driven processes such as hydrophobic recovery, a process where polymer chains reorient and low-surface-energy species migrate to the surface, thus potentially reversing the initial beneficial modifications of the RIP treatment.^27^ Such hydrophobic recovery could adversely affect the long-term endothelial compatibility of RIP-treated surfaces, as the initial improvements in wettability, surface chemistry, and topography subside.^28,29^ Furthermore, the material’s behavior over time is intricately linked to the specific RIP treatment parameters and storage conditions.^30^ The phenomenon of hydrophobic recovery underlines the necessity for ongoing research to elucidate its mechanisms and devise methods to counteract its effects. This understanding is crucial to ensuring that the biocompatibility and stability of cardiovascular biomaterials are maintained in the long term.

PVA is recognized for its nonthrombogenic and inert qualities, making it a viable hydrogel material for various biomedical applications, including small-diameter vascular grafts. Its suitability is further underscored by its mechanical properties—compliance and burst pressure—which can be finely tuned to align with those of native blood vessels.^31^ The versatility of PVA is evident in its ability to be synthesized with different cross-linkers, allowing for a range of chemical and mechanical characteristics to be achieved.^32,33^

Sodium trimetaphosphate (STMP), a food-grade cross-linker, is frequently utilized for hydrogel formation through a process known as phosphoesterification. This process creates a network of cross-links between phosphate groups and hydroxyl groups within the hydrogel, effectively forming a stable matrix.^31,34^ STMP is preferred over other cross-linkers like formaldehyde and glutaraldehyde, which have been associated with increased thrombogenicity and cytotoxicity of the resulting hydrogels. Notably, PVA cross-linked with STMP (STMP-PVA) and functionalized with aminated-fucoidan has demonstrated enhanced endothelialization and reduced thrombogenicity.^35^ The potential of STMP-PVA, including versions treated with RIP, to fulfill the material requirements for vascular grafts has been explored in numerous investigations, including studies conducted in nonhuman primate models.^34−37^ These studies indicate that RIP-treated PVA surfaces exhibit a higher affinity for ECs and a decreased accumulation of platelets and fibrinogen, particularly when compared to ePTFE grafts in thrombosis models without anticoagulation. Nonetheless, the long-term stability of the beneficial properties conferred to PVA by RIP treatment has yet to be characterized, a gap that is crucial to address for the clinical application of such promising biomaterials.

**Figure 1:**
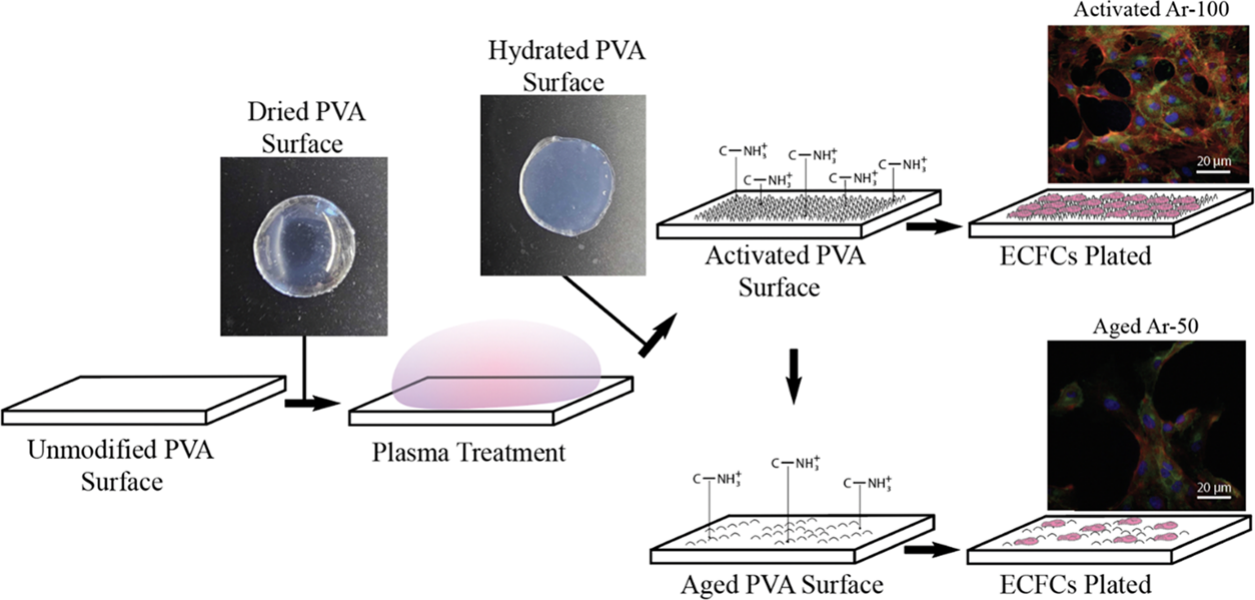
Schematic depicting the experimental procedure for quantifying ECFCs on activated or aged STMP-PVA surfaces. The hydrogel is cross-linked using sodium trimetaphosphate (STMP) and then subjected to reactive ion plasma (RIP) surface treatment in a dry state. This treatment introduces nanotopography and charged chemical species onto the surface, enhancing the endothelialization potential. Following the RIP treatment, the samples are hydrated for cell culture experiments. Over approximately 230 days of storage, the nanoscale surface roughness, charged chemical species, and endothelialization potential diminish. Representative fluorescence images of ECFCs cultured on activated Ar-100 and aged Ar-50 RIP-treated PVA are provided as insets. The images, captured at 20× magnification, are fluorescently labeled to indicate cell nuclei (blue), actin filaments (red), and VE-cadherin (green).

In the present study, we investigated the impact of RIP treatment on STMP-PVA using a selection of three precursor gases and two levels of RF power. The effects were monitored at two distinct time points: shortly after treatment within 14 days and at a prolonged interval of 230 days. The experimental conditions are depicted in Figure 1. To evaluate the changes induced by the RIP treatment, we employed X-ray photoelectron spectroscopy (XPS) for surface chemistry analysis, atomic force microscopy (AFM), and scanning electron microscopy (SEM) for topographical assessment. Furthermore, the potential for endothelialization was determined by measuring nuclear DNA 48 h following the seeding of ECFCs.

We posited that a surface textured by RIP treatment, exhibiting both roughness and charge, would enhance endothelialization. However, we also anticipated that these surface modifications might weaken over time. To the best of our knowledge, this is the first study to assess the persistence of the effects of RIP treatment on the surface properties of PVA and its subsequent influence on endothelialization. The findings from this research should significantly inform the development of RIP-treated biomaterials, including medical devices that are sterilized by using cold plasmas.

## 2 Materials and Methods

### 2.1 PVA Manufacturing

STMP-PVA was manufactured as previously described.^38^ Briefly, 15% (w/v) sodium trimetaphosphate (STMP, Sigma, St. Louis, MO) was added to aqueous PVA, followed by 30% (w/v) sodium hydroxide, and cured as films. A final concentration of 10% (w/v) aqueous PVA (Sigma, average MW 85–124 kDa, 87–89% hydrolyzed) was used for all hydrogel samples.

### 2.2 Reactive Ion Plasma Treatment

PVA samples were treated using a Plasma-Therm Batchtop VII apparatus (St. Petersburg, Florida). An RF power of 50 or 100 W with a DC bias of 370 V, pressure of 100 mTorr, and total gas flow rate of 50 sccm were used for all studies. Oxygen, nitrogen, or argon was used for the RIP treatments, and the samples were exposed to the RIP for 5 min. The sample nomenclature includes the type of precursor gas (Ar, N_2_, or O_2_) and the RF power at which the sample was treated. For example, argon treated at 50 W would be referred to as Ar-50. Samples were considered “activated” and used for characterization within 14 days of exposure to plasma. “Aged” samples were sealed and stored for approximately 230 days until characterization because 230 days from the initial treatment date was shown, in a similar polymer, to be the duration for hydrophobic recovery.^39^

### 2.3 Scanning Electron Microscopy

The PVA samples were mounted in a dry state on conductive double-sided carbon tape along with colloidal graphite to minimize charging. The mounted samples were then sputter-coated with Au/Pd at a ratio of 60:40 to form a 5 nm film. Images were collected using an FEI QUANTA 3D dual-beam scanning electron microscope at up to 50 000× magnification at either a 45 or 90° angle to the surface.

### 2.4 Atomic Force Microscopy

Untreated and RIP-treated PVA samples were measured in air using a Bruker Dimension Fastscan Bio Icon AFM at <14 days and at 230 days after treatment. PVA samples were trimmed with surgical scissors and adhered to glass slides using double-sided tape. The measurements were performed in Peakforce Tapping (PFT) Mode with Fastscan-C probes (spring constant = 0.8 N/m; end radius = 5 nm) on a Fastscan scanner. Samples were documented with 5 μm scans at 0.5–1 Hz and 512 × 512 resolution with a PFT frequency of 1 kHz. The peak force set point, amplitude, and gain were set to the lowest values, which enabled consistent tracking of the sample topology without loss of fidelity. Data processing and roughness quantification were performed using NanoScope Analysis 2.0 (Bruker Nano Surfaces, Billerica, Massachusetts). Four *R*_q_ values were determined for each of the aged and activated samples from distinct regions in the AFM scans. Prior to the roughness analysis, the scan data were flattened and plane fit, streak artifacts were removed, and a 3 × 3 median filter was applied to correct aberrant pixels resulting from noise.

### 2.5 X-ray Photoelectron Spectroscopy

XPS is a commonly used surface-sensitive technique that explores the chemical makeup of the top layer of a material up to a depth of 10 nm.^40^ XPS was used to determine the elemental composition of PVA before and after the RIP treatment. A spot size of 100 μm in diameter was used along with an electron flood gun for charge neutralization. The spectra were collected on a Versaprobe II (Physical Electronics, Chanhassen, Minnesota) at a takeoff angle of 45° with a monochromatic Al Kα source. One spot for each of the three different samples was collected for each scan. High-resolution scans were taken with a step size of 0.1 eV and a pass energy of 40 eV. The binding energy scales were calibrated to the CH_*x*_ peak at 285 eV in the C 1s region, with a linear background for peak quantification.

### 2.6 Endothelial Colony-Forming Cell Isolation and Culture

ECFCs were isolated from the peripheral blood of juvenile male baboons (Papio anubis) as previously described.^41−43^ Briefly, 50 mL of blood was collected in a 7% citrate solution via venipuncture before layering the blood on top of Histopaque-1077 (Sigma, St. Louis, MO) in centrifuge tubes in a 1:1 ratio. The tubes were centrifuged for 30 min at 500 G without a brake to isolate the mononuclear cells. Once mononuclear cells were collected, the cell suspension was washed with Hank’s buffer salt solution (HBSS, HyClone, Logan, UT) before centrifugation at 500 G for 10 min to form a cell pellet. Cells were then counted, resuspended in VascuLife VEGF Endothelial Medium (Lifeline Cell Technology, Frederick, MD) supplemented with 20% fetal bovine serum, and seeded onto tissue culture plates at a density of 20 million cells per well. Cells were placed in an incubator, and the medium was changed daily for the first 7 days, followed by every 3 days. Endothelial cell outgrowth colonies were allowed to develop for 2–4 weeks before ECFCs were collected via CD31 positive recognition using magnetic Dynabeads (Invitrogen, Carlsbad, California) and frozen for long-term storage.

### 2.7 Endothelial Colony-Forming Cell Quantification

ECFCs were cultured on 8 mm PVA samples as described previously.^36^ Quant-iT PicoGreen dsDNA Assay kits (Invitrogen, Carlsbad, CA) were used to quantify the number of cells present on the surface of the PVA punches 48 h after seeding and after each RIP treatment. PVA samples were first RIP-treated according to Section 2.2 and nontreated 48-well cell culture plates (Corning, Corning, NY) were coated with agarose prior to inserting PVA samples to block any contact between ECFCs and the bottom surface of the well after seeding. After allowing the ECFS to attach and proliferate for 48 h, the samples were washed thoroughly with PBS to remove any unattached cells and frozen overnight at −20 °C. The cells were then lysed with SDS, diluted in the TE buffer, and dsDNA was labeled using Quant-iT PicoGreen reagent (ThermoFisher, Waltham, MA). dsDNA from ECFC-seeded PVA was quantified as the fluorescence intensity from a standard curve of calf thymus DNA (Invitrogen, Carlsbad, California).

### 2.8 Immunostaining of Endothelial Colony-Forming Cells

Both activated and aged ECFC samples were subjected to an identical immunostaining process. The samples were first fixed in 48-well plates using 3.7% paraformaldehyde warmed to a physiological temperature. This was followed by a 10 min permeabilization phase with 0.1% TritonX-100. Image-iT FX Signal Enhancer (Invitrogen, Carlsbad, CA) was subsequently added to each well and given a 30 min incubation period to enhance fluorescence signal. F-actin was stained by administering Alexa Fluor 568 phalloidin (Invitrogen, Carlsbad, CA), which was diluted 1:200 in PBS, for 1 h. Next, nonspecific antibody binding was averted by blocking the wells with 10% goat serum in Buffer #1 for 30 min. Primary staining was carried out by adding VE-cadherin (Invitrogen, Carlsbad, CA), diluted 1:100 in PBS containing calcium and magnesium and 1% BSA, to each well and incubation for 1 h. Secondary staining was achieved using antimouse IgG1 Alexa Fluor 488, diluted 1:500 in PBS supplemented with calcium and magnesium. DAPI (Invitrogen, Carlsbad, CA) was then added at a dilution of 1:10 000 in PBS with calcium and magnesium and 1% BSA for a 5 min period for nuclear staining. Finally, the activated or aged PVA samples were delicately mounted onto glass slides using ProLong Gold Antifade (Invitrogen, Carlsbad, CA), to minimize photobleaching during subsequent microscopy. The samples were left undisturbed to cure at room temperature overnight in preparation for imaging.

### 2.9 Percent Confluence Calculation

To quantify the percent confluence of the ECFC cultures on the PVA samples, we performed the following calculations. Based on previous work,^36^ the untreated samples were confirmed to have no adhesion; therefore, any detected dsDNA was considered the background for all measurements. The surface area of a single ECFC was assumed to be 2000 μm^2^ and the cells were assumed to pack hexagonally.^44−46^ A mass of 7 pg of DNA per cell was used to calculate the number of cells on the STMP-PVA surface.^47^ First, the number of ECFCs per sample was measured by determining the total mass of dsDNA in the sample (*m*_total_) using the PicoGreen assay and dividing that value by the mass of dsDNA in a single ECFC (*m*_ECFC_).
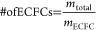


The number of ECFCs was then multiplied by the surface area of a single ECFC (SA_ECFC_) and divided by the cell packing efficiency (ε).
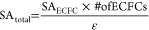


The result was a surface area representative of the total number of ECFCs on the sample (SA_total_), which was then divided by the total surface area of the substrate (SA_substrate_) to determine the % confluence.
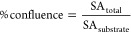


The value for the number of ECFCs on untreated samples was subtracted from the values for RIP-treated samples, as no cells were detected on the surface of untreated samples in this or previous studies using brightfield or fluorescence microscopy.

### 2.10 Electrostatic Force Analysis

Previous studies have shown that charged nanotopographic surfaces can exert considerable electrostatic forces on surrounding nanostructures.^48^ The modified electrostatic model developed by Lekner et al., which describes two uniformly surfaced charged cylinders,^49^ was used to gain insight into the forces that may influence the degradation mechanics of the RIP-treated PVA topography.

In this model, two uniformly charged cylinders with uniform dimensions (*q*_a_ = *q*_b_, *r*_a_ = *r*_b_) separated by a distance of *s* were considered. The bicylindrical coordinates *u* and *v* are introduced and mapped to Cartesian coordinates *x* and *y* via hyperbolic functions
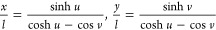
where *l* is the scale length defined
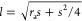


The force per unit length of the right-hand cylinder is given by
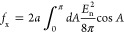


To expand this expression, angle *A* and bicylindrical coordinate *v* were related
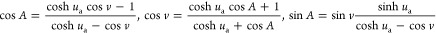

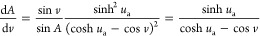


Substituting these expressions into the electrostatic force equation yielded
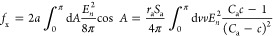
where *C*_a_ = cosh *u*_a_, *S*_a_ = sin*h u*_a_, and *c* = cos* v*. For uniformly charged structures, a three-term infinite series was derived to model the electrostatic force acting between cylinders
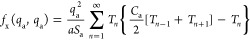
where *T*_n_ = *e*^–nu_a_^ tan* h* nu_a_. Nanostructure dimensions were determined from SEM images taken normal to the sample surface by using a custom MATLAB script and used in the derived expression to produce electrostatic force estimates.

### 2.11 Statistical Methods

SPSS 28 was used for all of the statistical calculations. Differences in group means of ECFC attachment between RIP treatments for either activated or aged samples were determined by using analysis of variance (ANOVA). First, we tested for normality and outliers in the dsDNA data, which was our dependent variable, for each RIP treatment (level). All data were normally distributed as groups (activated or aged) as well as at each level, except for aged O_2_-100 which had a statistically significant value for Shapiro-Wilk of 0.31. However, ANOVA is robust to variations of normality, so no adjustments or transforms were performed on the raw data. Two outliers were removed from Ar-50 aged and the untreated aged samples. Among groups, for aged samples, all data had equal variance, and for activated samples Levene’s test gave a significant value of 0.21, indicating unequal variances among levels. However, ANOVA is generally considered robust to the heterogeneity of variance if the largest variance is not more than 4 times the smallest variance, which was true for the activated samples. Furthermore, the general effect of heterogeneity of variance is to make the ANOVA less efficient. Therefore, any significant effects reported are still reliable. The data were further analyzed with Tukey’s post hoc test to determine differences among levels compared to untreated samples within groups using a pairwise comparison. A *p*-value of < 0.05 was used to determine significance among levels within groups. Differences between groups within a level were tested using a one-sided paired *t* test. AFM results are reported as level means and standard deviations. Level means were compared pairwise using a one-sided paired *t* test and within groups using ANOVA with Tukey’s post hoc. All XPS data indicating the charged and uncharged nitrogen species were evaluated using a paired two-sided *t* test between groups and levels were not tested for XPS data.

## 3 Results

### 3.1 Scanning Electron Microscopy Images

**Figure 2:**
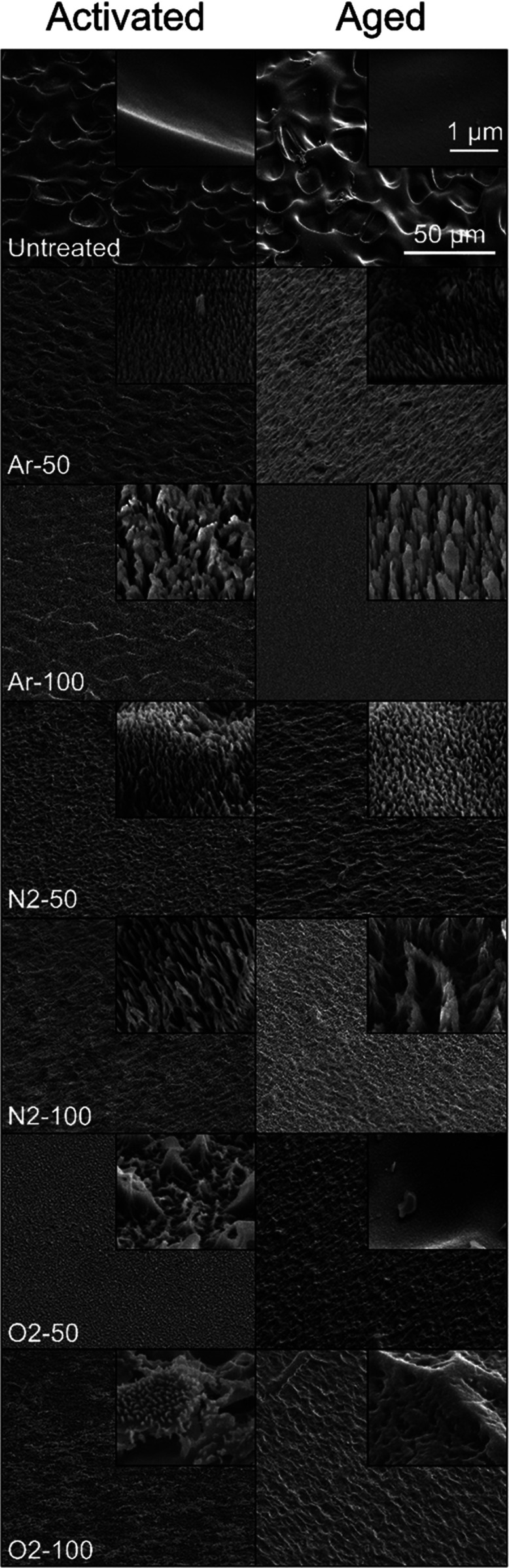
Scanning electron micrographs of untreated, activated, and aged PVA samples at 45° angle to the surface. The top row shows images of untreated PVA, the left-hand column shows images of activated PVA, and the right-hand column shows images of aged PVA. The order of RIP treatments from top to bottom is Untreated, Ar-50, Ar-100, N_2_-50, N_2_-100, O_2_-50, and O_2_-100. Larger images were collected at 1000× magnification with a 50 μm scale bar, and inset images were taken at 50 000× magnification with a 1 μm scale bar. RIP treatments impart nanohairs on the surface of the activated samples that are diminished in the aged samples. The qualitative character of the topographic surface modifications of activated and aged samples depends on the ion source gas and the power of the RIP treatment.

Images of the untreated, activated, and aged samples collected by using SEM at 45° relative to the surface are shown in Figure 2. Images of untreated PVA are shown in the top row. Images of activated PVA are in the left column and aged samples are in the right column. From top to bottom, the order of RIP treatments is Untreated, Ar-50, Ar-100, N_2_-50, N_2_-100, O_2_-50, and O_2_-100. The larger images have a 50 μm scale bar and were collected at 1000× magnification. Inset images have a 1 μm scale bar and were taken at 50 000× magnification. The underlying microscale porous features observed in the untreated PVA (top row) appear to remain in the activated samples, which also showed additional nanotopographic features and therefore hierarchical structures (left-hand column). The same microstructures are visible, but less pronounced in the aged samples, while the magnitude of the nanostructures diminished significantly or disappeared completely in the aged samples (right-hand column).

### 3.2 Atomic Force Microscopy-Based Analysis of Surface Topography

**Figure 3:**
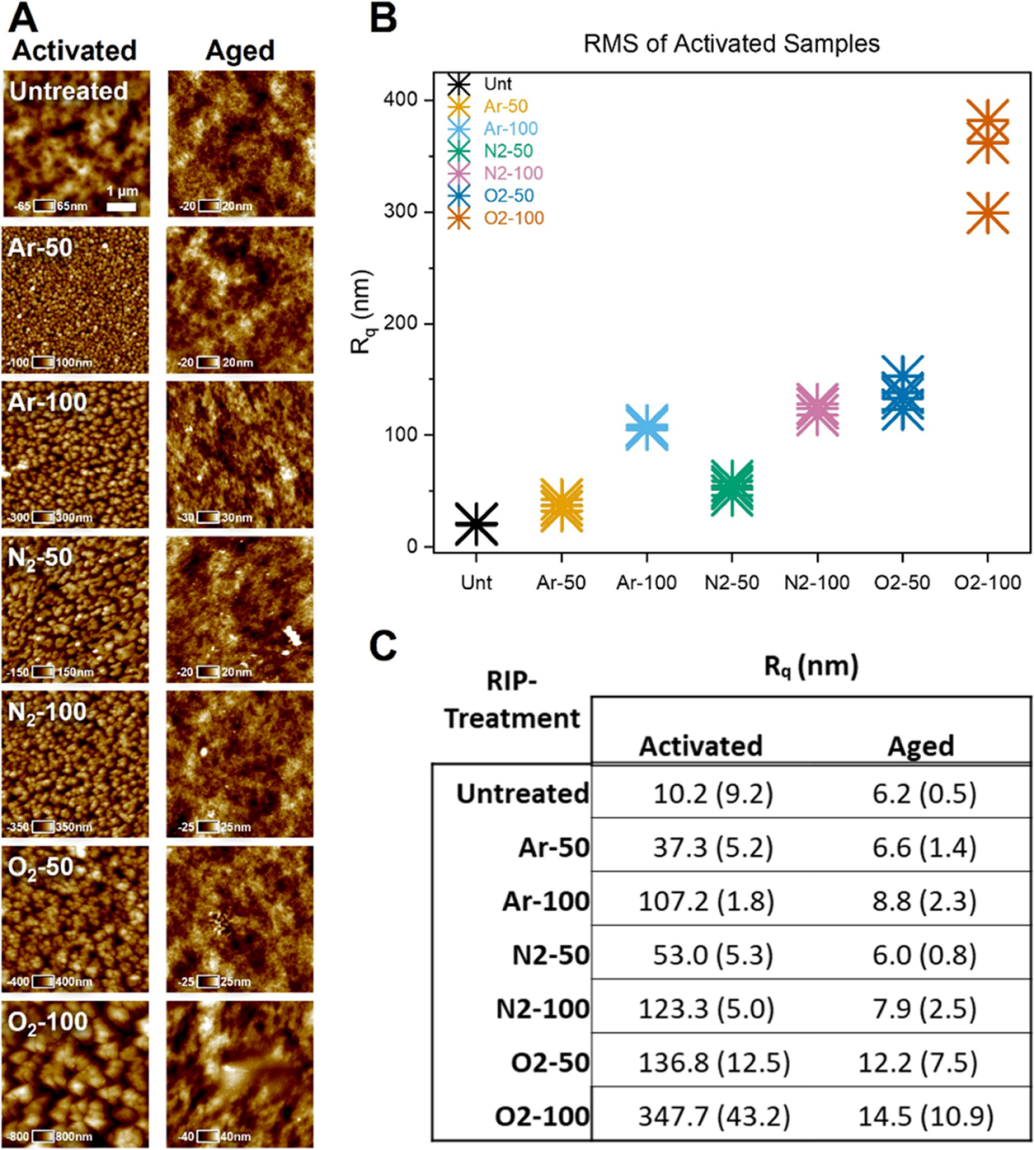
(A) Atomic force micrographs of activated and aged PVA samples for each RIP treatment and the untreated PVA. An in-plane scale bar of 1 μm is shown in the micrograph of untreated PVA, with orthogonal height values specific to each image. (B) Plot of the root-mean-square roughness (*R*_q_) for each of the activated samples and the untreated polymer (*n* = 4). Activated samples at both RF powers were significantly different from the untreated samples with a one-sided paired *t* test (*p* < 0.05). (C) *R*_q_ values for activated and aged samples presented as the mean ± standard deviation. All activated samples returned to the approximate roughness of untreated PVA after aging for all RIP treatments, and were significantly different from activated samples according to ANOVA and Tukey’s post hoc test (*p* < 0.05).

AFM was used to characterize the topographies of the untreated and RIP-treated PVA surfaces. Surface scans revealed differences in surface roughness, which varied according to the precursor gas and RF power. The results of topographical characterization are shown in Figure 3. The root-mean-square roughness, *R*_q_, of the activated samples was approximately one order-of-magnitude greater than that of the aged samples of the same treatment. The observed differences in roughness for the recently activated samples required more scans of the surface to properly account for heterogeneity within and between the samples. In contrast, the aged samples exhibited surfaces that relaxed almost to the baseline roughness observed in untreated PVA, with relative homogeneity within and between the samples. Untreated and activated samples at both powers were found to be significantly different (*p* < 0.05) and consistent across all precursor gases. The N_2_ and Ar-activated samples exhibited similar levels of roughness at both RF powers, but the O_2_ samples were consistently rougher at both powers. Morphologically, the activated and aged O_2_-100 samples appeared to be qualitatively different from those of the other treatments within their respective groups. Using a two-tailed *t* test, 100 W samples were determined to have significantly different *R*_q_ values than those of the samples treated at 50 W (*p* < 0.05). Increased roughness at higher RF powers was observed for both activated and aged samples.

### 3.3 X-ray Photoelectron Spectroscopy Spectral Analysis of Surface Chemistry

**Figure 4:**
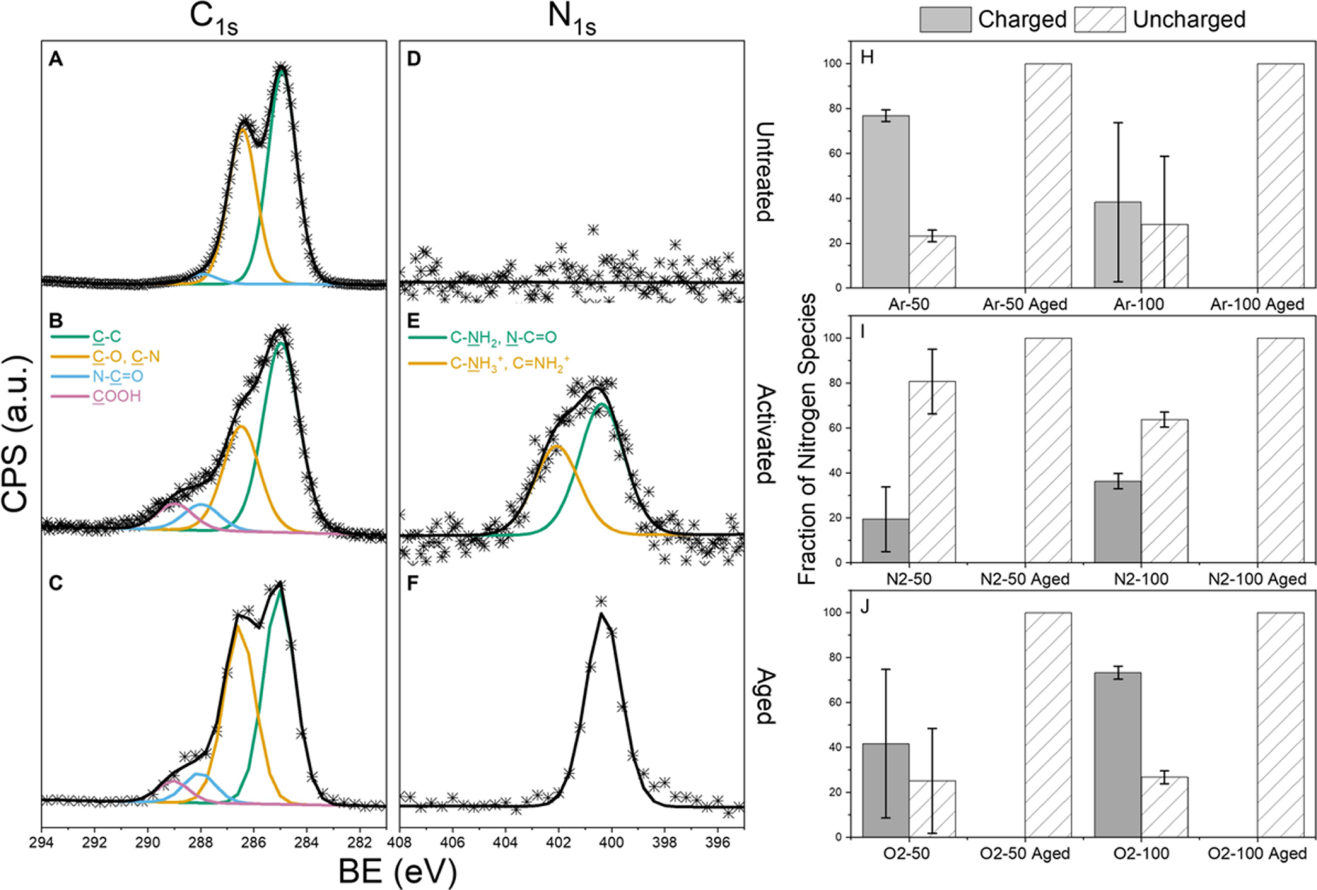
(A–C), (D–F) (Left) High-resolution XPS scans of the C 1s and N 1s regions of PVA with N_2_-100 RIP treatment. The top row (A, D) represents unmodified PVA, the middle row (B, E) shows activated PVA after N_2_-100 RIP treatment, and the bottom row (C, F) shows aged PVA 230 days after N_2_-100 RIP treatment. The C 1s scans remained relatively unchanged between the activated and aged samples. In the N 1s region, a charged species of nitrogen was present in the activated sample but not in the aged sample. (H–J) (Right) The fraction of nitrogen species for each RIP treatment shown as either charged or uncharged nitrogen. The top row (H) shows the Ar-treated samples; the middle row (I) shows the N_2_-treated samples, and the bottom row (J) shows the O_2_-treated samples. The left side bar (solid gray) of each panel shows the activated samples, and the right bar (diagonal hatch) shows the aged samples. No charged nitrogen was detected in any aged samples. Measurements were conducted with a 100 μm diameter spot size, and three samples were measured for each treatment to calculate variance; the error bars indicate the standard deviation.

XPS was used to probe the changes in the surface chemistry of untreated, activated, and aged PVA. The N2-100 sample was representative of the chemical species observed in the other five samples and shown in Figure 4. High-resolution scans collected in the C 1s region of the N2-100 sample revealed the presence of four species under the C 1s peak envelope. Untreated PVA contains two of the four species, C–C (285 eV) and C–O/C–N (286.5 eV).^50^ Untreated PVA contains equal parts (50:50) of C–C and C–O; however, the C–C component likely contains adventitious carbon, which would explain the higher than expected C–C (285 eV) peak. High-resolution scans of the RIP-treated, activated, and aged samples in the C 1s region (Figure 4B,C) revealed the addition of two new species: an amide bond, N–C=O (288 eV), and a carboxylic acid group (289 eV).

There was no peak in the high-resolution spectra in the N 1s region for untreated PVA, as shown in Figure 4D. For activated PVA, the spectra in the N 1s region are shown in Figure 4E and contain two distinct peaks at 400.2 and 401.9 eV. The first of the two peaks corresponds to C–N species, such as C–NH_2_^51−54^ or N–C=O,^54−58^ which is consistent with the species observed after plasma exposure.^59,60^ The second peak centered at 401.9 eV consists of charged quaternary nitrogen.^52−54,61^ Although the charged functional groups likely include C–NH_3_^+^ and C=NH_2_^+^, the overlap of the charged quaternary groups does not allow for the assignment of a single quaternary species. The N 1s spectra of the aged samples (Figure 4F) contained a single peak at 400.2 eV.

The fractions of charged and uncharged species present in each treatment are shown in Figure 4H–J. Quaternary or charged nitrogen species were observed only in the activated samples. The error bars representing the three different scans show a wide distribution of charged and uncharged species on the phosphors of the O_2_-50 and Ar-100 samples.

### 3.4 Percent Confluence of Endothelial Colony-Forming Cell Cultures

**Figure 5:**
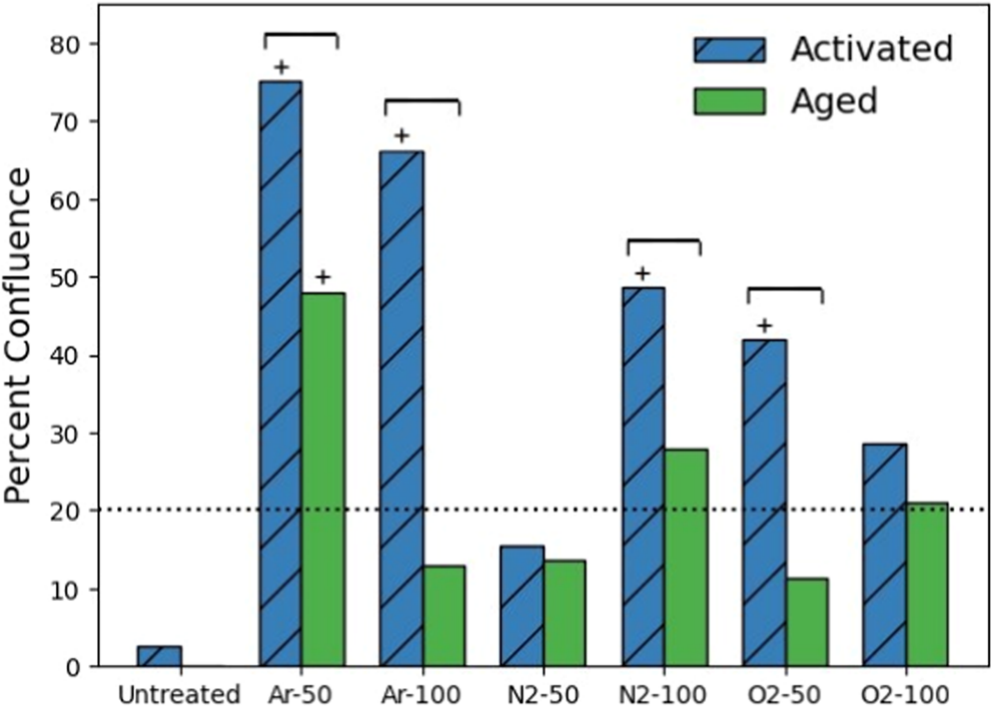
Bar graph of the percentage of endothelial colony-forming cell confluence on RIP-treated PVA samples 48 h after seeding. The blue bars with the diagonal hatch represent the activated samples, located left of center, while the green bars represent the aged samples, located right of center. The dotted line indicates the cell seeding density. Brackets represent significant differences between activated and aged samples for a single RIP treatment level using a one-sided paired *t* test. Bars marked with a “+” indicate a significant difference from untreated PVA according to ANOVA with Tukey’s post hoc (*p* < 0.05). After aging of the RIP-treated samples, the percentage of endothelial cell confluence decreased for all treatments compared to the activated samples.

The percent confluence of the ECFCs on plasma-treated PVA is shown in Figure 5. The percent confluence of ECFCs attached to the activated samples for O_2_-50, O_2_-100, Ar-50, Ar-100, N_2_-50, and N_2_-100 was 42, 29, 75, 66, 16, and 49%, respectively. The percent confluence of ECFCs attached to aged samples for O_2_-50, O_2_-100, Ar-50, Ar-100, N_2_-50, and N_2_-100 was 11, 21, 58, 13, 14, and 28%, respectively. The activated and aged samples were found to be significantly different by ANOVA (*p* < 0.05). The activated O_2_-100 and N_2_-50 samples were not significantly different from the untreated samples. In the aged group, only Ar-50 showed a significant increase in attachment compared to the untreated group.

### 3.5 Electrostatic Force Model

**Figure 6:**
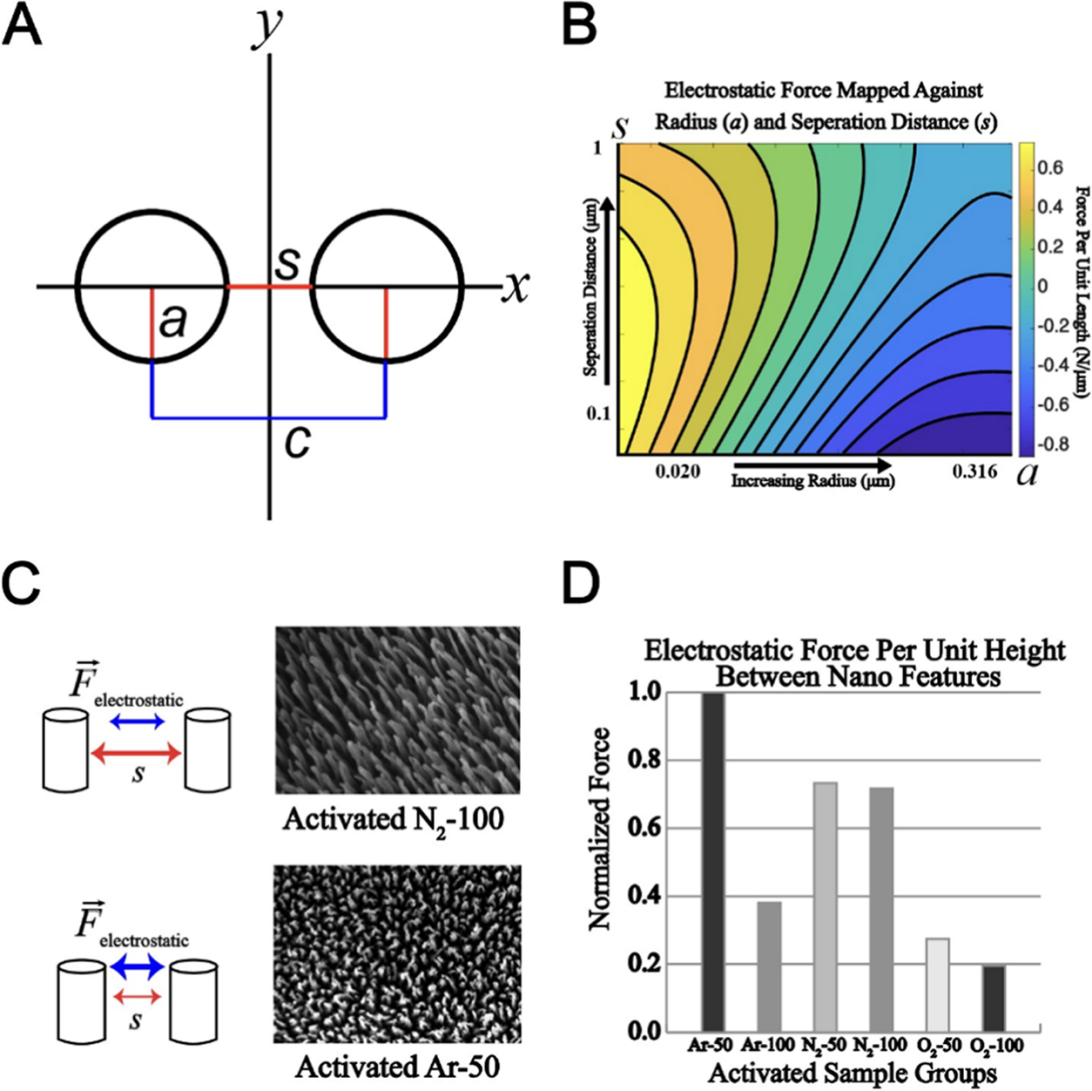
Analysis of electrostatic forces within RIP-etched STMP-PVA nanohairs. (A) Uniform-charged cylinder model representing the nanohairs etched into the STMP-PVA. (B) Log-scale plot showcasing the forces generated by nanohairs, based on their radius (a) and separation distance (s). (C) SEM images captured normal to the substrate surface, used for model parameter calculation for each sample type. (D) Bar graph representing the estimated electrostatic forces across samples, normalized by the maximum predicted value (Activated Ar-50), to facilitate comparisons of the force for each RIP treatment type.

Figure 6 contains the details of the analytical model used to estimate the electrostatic forces across activated samples. The geometry used to calculate the forces is shown in Figure 6A. The electrostatic force as a function of separation distance and pilar radius is shown in Figure 6B. Figure 6C,F shows the SEMs used to calculate the separation distance and pilar radius for Ar-50 and Ar-100, respectively. Figure 6D shows the calculated values for electrostatic force between nanohairs normalized by the maximum force within the data set, the Ar-50 treatment. Based on the derived model, the groups exposed to nitrogen treatment exhibit force magnitudes that were approximately 30% lower in comparison to the Ar-50 group. Additionally, the Ar-100, O_2_–50, and O_2_–100 samples have significantly reduced electrostatic forces of approximately 60, 70, and 80%, respectively.

## 4 Discussion

### 4.1 Endothelial Colony-Forming Cell Coverage

Regardless of the precursor gas, RIP treatment of PVA was shown to support ECFCs at 48 h compared with untreated PVA and to different degrees for different powers and precursors. The percent confluence of ECFCs of active vs aged samples supports the existence of the shelf life of the RIP-treated material. Although RIP-treated PVA has been shown to support cell attachment, spreading, and proliferation,^36,62^ to the best of our knowledge, this is the first study to demonstrate time-dependent changes in the ECFC attachment to RIP-treated PVA. This was demonstrated by comparing the activated samples, which showed increased endothelialization, with their corresponding aged samples, which adhered significantly fewer cells, except the O_2_-100 and N_2_-50 samples. These data suggest that there is a period after treatment in which the material can successfully facilitate ECFC attachment. Adherence of endothelial cells to the material is critical for endothelialization and, therefore, for its long-term performance and clinical acceptance as a potential candidate for SVGs.

It is well established that treating polymers with RIP leads to changes in the surface chemistry of the materials as well as changes in roughness, surface energy, and wettability.^63−65^ It is difficult to determine whether any of these changes in the material properties alone are responsible for the increased cell affinity. ECFC viability after attachment depends on specific cell-surface integrin interactions with ECM proteins along with the associated signaling networks and is therefore not likely to be reducible to a single determinant surface property. However, alteration of the surface chemistry or roughness of a biomaterial can lead to changes in its ability to adsorb ECM proteins from the surrounding microenvironment or encourage a cell to deposit its own matrix proteins.^63^ Cells can interact directly with a surface briefly through weak interactions in the absence of ECM proteins but will undergo apoptosis within 24 h if vital inter- and intracellular signaling has not been initiated.^63,64^ Cell viability studies, expression of adhesion molecules, measurements of deposited proteins, and characterization of EC behavior such as attachment, migration, and proliferation should be performed in the future to isolate the specific binding mechanism of ECFCs to RIP-treated surfaces and their phenotypic consequences as well as to determine the effect of both activation by RIP and aging on the endothelialization potential of PVA. Additionally, Future studies could consider the use of SEM images of endothelialized samples to further explore the colocalization of geometric features on RIP-treated PVA and cellular substructures such as lamellipodia, filipodia, and focal adhesions.

### 4.2 PVA Surface Roughness

The RIP treatments had a significant impact on the surface roughness at each power level compared to the untreated sample. This was most prominent for the O_2_ treatments, which might have resulted in an increased interaction between the plasma and native oxygen within the polymer. The decrease in the surface roughness over time to near-baseline levels for the aged samples can be explained by hydrophobic relaxation, which results from a thermodynamically unstable surface composed of hydrophilic groups that are entropically driven into the bulk of the polymer. Relaxation reduces the roughness of the surface and shifts the chemical groups initially formed on the surface to the bulk of the material. This outcome has implications for the broader use of plasma-treated polymers in medicine, as it supports the idea of shelf life. The shelf life of RIP-treated polymers designed to encourage endothelialization represents a period during which the polymer actually promotes EC adherence. During this period, the treated polymer has rougher surface topography and functions differently than samples treated with RIP but given time to age. This idea is important for the use of PVA as a vascular graft material and its endothelializability. Most likely, other important characteristics of biomaterials such as their thrombogenicity will change as a roughened and charged surface relaxes, although that was not specifically tested in this study.

### 4.3 Charged Functional Groups

The disappearance of the peak at 401.9 eV in the aged sample indicated that the quaternary species previously observed in the activated samples were no longer present, suggesting that at some point between the initial treatment and the data collection that occurred 230 days after the treatment, the charged species were neutralized or transported off the surface. The results from the XPS high-resolution data in the N 1s region for N_2_-100 are representative of the other plasma treatments in terms of observable charged species. The only observed charged species were the quaternary nitrogen groups on the surface. It has been shown in the literature that nitrogen is not incorporated beyond the surface of RIP-treated polymers.^27,66^ The presence and duration of charged nitrogen species indicate the period in which the polymer is in its optimal state to facilitate endothelialization. More studies may reveal the optimal length of time and storage conditions for such materials to be ideal for use as vascular grafts to enable the greatest potential for endothelialization. The charged species observed in all treatments were found in only activated samples. No quaternary nitrogen was found in aged samples across the different treatments. This finding supports the idea that charged species are neutralized over time and contribute to the adhesion of ECFCs either through the direct binding of integrins or through the electrostatic attraction of binding proteins.

### 4.4 Nanostructures

The formation mechanism of the observed nanostructures shown in Figures 2 and 3 can be explained by ion etching of the insulating PVA. Previous work has shown that ion etching of an insulating polymer that considers charging leads to the formation of high-aspect-ratio features.^67^ This work suggests that the impinging ions are deflected from the peaks and enhance the etch rate of the sidewalls of the features to form high-aspect-ratio structures, which are described as nanohairs produced by anisotropic etching.^68,69^ The nanohairs were confirmed in the AFM scans and are shown in Figure 3. However, the structure was more easily observed in the SEM images in Figure 2, which highlights the changes in the surface at the nano- and microscales for untreated PVA followed by RIP treatment. SEM images also provided evidence of material ablation and redeposition in the O_2_-50 and O_2_-100 samples, which is a phenomenon known to occur when RIPs are generated using DC bias.^70^ Therefore, redeposition is another important phenomenon to consider when designing RIP-treated vascular graft materials in addition to hydrophobic recovery, nanotopographic relaxation, and quaternary nitrogen neutralization or transport.

The interplay of near-field and electrostatic forces is known to significantly influence the geometric features at the nanoscale, especially in the context of charged surfaces.^49^ In our study, these forces might have played a substantial role in the observed degradation mechanics of nanotopography across all RIP treatments, especially with the gradual dissipation of quaternary species over time.

To further understand these dynamics, we turned to Whipple’s modified model, which describes the electrostatic forces between two uniformly charged cylinders. When applied to our RIP-treated PVA samples, this model suggested that Ar-50, Ar-100, N_2_-50, and N_2_-100 treatments could generate the most significant repulsive electrostatic forces between nanohairs. This is primarily due to their respective widths and separation distances. Notably, our model predicted significant differences in electrostatic forces among the activated samples, particularly within the O_2_-50 and O_2_-100 groups.

These findings underscore the extent to which different RIP treatments can influence surface topographies and, thereby, modulate the electrostatic interactions between nanostructures. They also provide crucial insights into how the geometry might be involved in the degradation kinetics of a roughened surface. For instance, we observed that PVA subjected to N_2_ and Ar RIPs demonstrated reduced relaxation over time. This suggests a potential correlation between the treatment-induced geometry and certain mechanisms that slow surface topography degradation.

Although our model’s main utility lies in suggesting a mechanism for the observed degradation of nanohairs on RIP-treated PVA, it also allows us to establish an indirect link between near-field electrostatic forces and endothelialization. This is achieved by probing the potential effects of these forces on the degradation of nanofeatures, which are known to influence endothelialization.^17^ Nonetheless, this is a complex area that warrants further investigation, both to clarify the underlying mechanisms contributing to these observed differences in force magnitudes and to understand how these forces may correlate with the instability of surface topographies.

## 5 Conclusions

This study characterized the effect of RIP treatment and storage time on STMP cross-linked PVA for potential use as small-diameter synthetic vascular graft materials for treating cardiovascular disease. RIP treatment introduces nanohairs and charged nitrogen species onto PVA which enhances its endothelializability. AFM and SEM analyses of treated surfaces indicated a rougher surface in the activated samples after plasma treatment that relaxed to a smoother surface over time. SEM images showed the presence of high-aspect-ratio features, which were the main contributors to the surface roughness observed in AFM, as well as the redeposition of ablated PVA in samples treated with O_2_ RIPs. XPS revealed the addition of new charged functional groups during the treatment, which disappeared over time. We proposed a model that suggests that the charge of RIP-treated PVA affects the physical degradation of nanohairs and vice versa. The increase in the endothelialization potential of activated samples compared to untreated PVA correlates with a rougher surface and the presence of charged functional groups. After aging the samples for 230 days, the smoother and less charged surfaces exhibited a decrease in endothelialization compared with the recently activated surfaces and untreated PVA. The chemical and physical changes in the PVA surface resulting from RIP treatment are known to promote tissue regeneration and previous studies have shown that PVA treated with N_2_-100 plasma is less thrombogenic than the current clinical standard synthetic vascular graft material: ePTFE. However, our results also suggest that RIP modifications of PVA are not permanent and appear to relax over time. If this time-dependent phenomenon is generally applicable to plasma-treated polymers, as this study and the literature suggest, it could have major implications for the broader usage of polymeric materials that receive RIP treatment, including the durability of plasma modifications intended to sterilize medical devices, improve biocompatibility, or improve cell adhesion.

## Data Availability

The data sets generated for this manuscript will be made available upon reasonable request.

## Notes

This study does not perform any experiments on animals. All collection of primary cell samples took place at the Oregon National Primate Research Center (ONPRC) and approved by the Institutional Animal Care and Use Committee when appropriate. Baboons were cared for at ONPRC according to the “Guide to the Care and Use of Laboratory Animals” prepared by the Committee on Care & Use of Laboratory Animals of the Institute of Laboratory Animal Resources, National Research Council (International Standard Book, Number 0-309-05377-3, 1996, the United States).
